# Anticipated impact of stem cell and other cellular medicine clinical trials for COVID-19

**DOI:** 10.2217/rme-2021-0025

**Published:** 2021-06-11

**Authors:** Mina Kim, Paul S Knoepfler

**Affiliations:** ^1^Department of Cell Biology & Human Anatomy, School of Medicine, University of California, Davis, 1 Shields Ave, Davis, CA 95616, USA; ^2^Institute for Pediatric Regenerative Medicine, Shriners Hospital for Children, 2425 Stockton Blvd., Sacramento, CA 95817, USA

**Keywords:** cellular medicine, clinical trials, COVID-19, MSCs, regenerative medicine, stem cells

## Abstract

**Aim:** There is a critical need for safe and effective treatments for COVID-19. One possible type of treatment is cellular medicine such as stem cell therapy, but its potential is unclear. Here, our aim was to assess the potential impact of the many cellular medicine trials for COVID-19. **Materials & methods:** We collected and analyzed data for defined criteria from trial registries. **Results:** Our data suggest that relatively few of these COVID-19 trials will produce high-level evidence, but that on average they may be somewhat more rigorous than typical cell therapy trials unrelated to COVID-19. **Conclusion:** Most COVID-19 cellular medicine trials have relatively low potential for rapid, concrete impact. We discuss the findings in the context of the cellular medicine field overall.

Since the beginning of the coronavirus pandemic, the number of cases has rapidly risen from a starting group of 41 known individuals on 12 January 2020 in Wuhan, China to more than 140 million confirmed cases worldwide throughout the course of just over a year. While the implementation of several new vaccines starting in late 2020 and still being rolled out in 2021 brings concrete hope, there is a critical need for safe and effective treatments for COVID-19. Treatments will be needed because of the slow rollout of the vaccines, uncertainties about how long immunity post infection or vaccination will last, the evolution of new mutant strains of severe acute respiratory syndrome coronavirus 2 (SARS-CoV-2) and the fact that billions of people will likely remain unvaccinated for years to come.

One emerging potential kind of COVID-19 treatment is cellular medicine such as stem cell therapy [1]. Dozens of new trials of cell therapies for COVID-19 have generated major attention and valuable resources are being devoted to these clinical studies, but at present the potential for meaningful impact from this line of clinical research is unclear. Although publications in 2020 and early 2021 on small clinical studies or case reports [2,3] generated some early optimism, there is relatively little rigorous data so far to support the widespread adoption of clinical use of any cellular therapies and more specifically stem cell-based interventions for COVID-19. There has been concern that because of the urgent nature of the pandemic that COVID-19 trial standards overall and those of cellular COVID-19 therapies specifically could be lowered, as compared with nonpandemic-related trials [4,5]. Another challenge consists of what are broadly termed as ‘unproven stem cell clinics’, which have been increasing in number and marketing unapproved cell therapies for many conditions [6]. Some of these clinic firms also have begun new marketing centered on COVID-19 [7], putting patients at risk and sparking increased activity by the US FDA and Federal Trade Commission in the USA [8].

More broadly, cellular medicines have great promise for the future treatment of a variety of diseases [9], but to date few cellular medicines for specific applications have received FDA approval in the USA. Both safety and efficacy standards for cellular medicines need to be considered by regulators like the FDA, which also need to encourage innovation in this space, together presenting major oversight challenges [10–12]. While a few other cellular medicine trials have led to approvals abroad, there is a general sense that the potential of this field is mostly ahead of us. Still, particularly in the area of immune modulation using mesenchymal stromal/stem cells (MSCs), the approved uses of MSCs for a few conditions including for certain groups of patients such as children with specific forms of graft-versus-host disease (GvHD) [13], have raised optimism both for a wider range of future regulatory approvals and for the subsequent potential positive impact of these kinds of cellular products on patients.

The rationale for use of MSCs for GvHD and other diseases characterized by harmful, elevated immune system activity is based on the observation that these cells can in some cases reduce elements of such immune activity [1]. This immunomodulation rationale and the GvHD clinical research together have been the driving force in launching many cellular medicine clinical research efforts aimed at COVID-19, where a substantial part of the morbidity and mortality is thought to be due to overactive immune responses including cytokine storms [14]. The potential impact of these cellular medicine efforts on the pandemic remain unclear.

Here, to address this gap we systematically analyzed clinical trials that reported using cellular products including MSCs for the treatment of COVID-19. We collected data from their listings on ClinicalTrials.gov and the Chinese trial registry, Chictr.org. We found that with a few notable exceptions, most of these trials are very small although most are later than Phase I. Also, while many include randomization, relatively few include double blinding or placebo controls, raising concerns about lack of conclusive impact from the data to be collected. Although the urgency of the pandemic could have catalyzed a large number of weak cell therapy trials (relatively speaking as compared with other cell therapy trials unrelated to COVID-19), we found that, at least as compared with two control sets of non-COVID-19-related cellular medicine trials, the COVID-19 cellular medicine trials generally had higher anticipated impact. Overall, the investigation of cellular medicines for COVID-19 continues and there is hope that the most rigorous of the trials may yield conclusive results and possible future clinical impact. However, the fact that only a minority of clinical studies we identified met the selected design standards raises the possibility of mostly only very preliminary results overall for an extended period of time. Our findings also have broader bearing on the design features of cellular therapy and specifically stem cell clinical trials overall.

## Materials & methods

We collected data from the cell therapy listings on two clinical trial registries, focusing on ClinicalTrials.gov and Chictr.org, based on the rationale that these are among the two most widely used trial databases. We searched on these registries with the target disease listed as ‘COVID-19’ along with the exact terms ‘MSC’ and ‘stem cell(s)’ for ClinicalTrials.gov and ‘mesenchymal’ and ‘stem’ AND ‘cell(s)’ for Chictr.org. The first searches for these keywords were completed on 10 July 2020 for ClinicalTrials.gov and on 4 and 8 August 2020 for Chictr.org. The somewhat different search terms were used for the two databases because of the idiosyncrasies of Chictr.org, which led to some search terms producing either no results or unrelated results on that database.

Data from control sets of non-COVID-related cellular therapy trials were also collected and analyzed including for trials related to arthritis and trials with no particular disease criteria specified other than being non-COVID-19 related. The rationale for the use of the arthritis cell therapy group as a control is that as a relatively established area of clinical investigation in the cellular therapy field it presents a helpful, large cohort for comparison to the broad area represented by the ‘no particular disease’ group. For the no-disease control group, because there were far >57 trial listings on ClinicalTrials.gov, the results of the search were randomized and separated into three different sets of 57. Some studies in these randomly selected groups ended up being noninterventional or COVID-19 related, so those were then excluded prior to analysis. We note that the random nature of the no-disease control dataset probably hides some relevant heterogeneity.

We also separately identified COVID-19 trials using natural killer (NK) cells as well. Search terms for NK cells were ‘NK cell(s)’ for both ClinicalTrials.gov and Chictr.org. Data for NK cell trials were collected on 10 July 2020 from ClinicalTrials.gov and on 9 August 2020 from Chictr.org.

Search results data were combined into a working database. The database was manually curated by the two authors to exclude both duplicates and observational listings that did not involve administration of a cell therapy-related product into COVID-19 patients, or in the case of the control groups, any trials that did involve COVID-19. For data presentation in tables actual numbers and percentages were rounded up to no more than three significant digits.

## Results

### Stem cell & ‘MSC’ clinical trials for COVID-19 results

On ClinicalTrials.gov, we identified 57 COVID-19 trials that were relevant to our search keywords for stem cell or MSC-related cell therapy studies, but two were then excluded since they were subsequently determined to be noninterventional. Within the resulting dataset of 55 trials, the number of participants ranged from five to 400, with 54 being the mean (Table 1). Most of the trials, 49 of the 55 listings, plan to utilize MSCs in some way. In terms of anticipated impact based on design features of this group of COVID-19 trials, 67.3% were randomized and 32.7% were at least double blinded. We found that 47.3% were placebo controlled. Only 30.9% met all three criteria of being randomized, at least double blinded and placebo controlled.

**Table 1.** Trial overall characteristics

Trial type	N of trials	Mean # participants	Randomized	At least double blinded	Placebo controlled	All three
COVID	55	54.0	37 (67.3%)	18 (32.7%)	26 (47.3%)	17 (30.9%)
COVID China	15	48.4	9 (60%)	1 (6.7%)	1 (6.7%)	1 (6.7%)
Non-COVID	38.7	40.7	19.7 (50.8%)	8 (20.5%)	10.33 (26.6%)	5.67 (14.5%)
Arthritis	55	144.6	32 (58.2%)	17 (30.9%)	12 (21.8%)	8 (14.6%)
NK	5	45.6	3 (60%)	1 (20%)	0 (0%)	0 (0%)
NK China	4	42.5	4 (100%)	0 (0%)	0 (0%)	0 (0%)
All COVID	79	51.8	53 (67.1%)	20 (25.3%)	27 (34.2%)	18 (22.8%)

NK: Natural killer.

Of the 15 unique, relevant stem cell or MSC-related cell therapy COVID-19 trials that were on the Chinese clinical trial registry, the range of participants was nine to 200, with 48.4 participants being the mean (Table 1). Moreover, 12 of the 15 trials involved MSCs, and one separate trial was found to be a combination of ruxolitinib and MSCs. While 60% were randomized trials, only 6.7% were double-blinded trials, and just 6.7% placebo-controlled trials. Only one trial (6.7%) met the most rigorous standard of being randomized, double blinded and placebo controlled.

### Characteristics of the control group of stem cell clinical trials not specifically related to COVID-19

As a control, we collected three sets of 57 ClinicalTrials.gov stem cell-related trials for which we did not specify a particular disease. The key search terms were kept consistent as ‘MSC’ and ‘stem cell’ for comparison to the COVID-19 ClinicalTrials.gov group. After collection of these trial groups, we eliminated COVID-19-related and observational trials, yielding somewhat smaller final groups for analysis (mean of 38.7 trials for the three groups). Calculations were conducted with each respective set and then means of the three control no-disease, non-COVID-19 groups were calculated. Non-COVID-related trial sets had a mean of 40.7 participants. A mean of 50.8% of control trials was randomized, 20.5% were double blinded, 26.6% placebo controlled and 14.5% met all three criteria (Table 1).

The second control group consisted initially of 57 arthritis-related, cellular medicine trial listings from ClinicalTrials.gov, with two observational trials being excluded this yielded a final group of 55 trials for analysis. The arthritis trials stood out for having a much larger number of participants with a mean of 144. This control arthritis group had a mean of 58.2% randomized trials, 30.9% were double blinded, 21.8% placebo controlled and 14.6% met all three criteria (Table 1).

### NK cell clinical trials results

We found that NK approaches are only more rarely being explored as a treatment for COVID-19 as compared with MSCs and stem cells. Only eight trials using the search terms ‘NK cell(s)’ and ‘covid19’ were found on ClinicalTrials.gov. Three of these eight trials were noninterventional, observational studies that did not infuse patients with NK cells but used NK cells as a marker for a patient’s condition. The mean number of participants overall was 45.6. None of the trials met all three criteria of being randomized, double blinded and placebo controlled.

Even fewer NK cell trials for COVID-19 were found on the Chinese clinical trial registry. Four total trial listings resulted from our search, with a range of 20–90 participants and a mean of 42.5. All of the clinical trials were randomized, three were intravenous interventions and none were placebo controlled.

### Comparing data on trial sizes

Trial size is another potential predictive indicator of anticipated rigor. As mentioned in the sections above, we collected data on the sizes of trials in each group. While there were some differences, it is mostly unclear if they are meaningful. The one group that stood out in size was cellular therapy trials for arthritis, which had two to threefold more participants than the other groups. This large mean size could be because of arthritis being a target disease for treatment with cellular therapies for more than a decade or because of the large number of arthritis patients readily available for potential enrolment in trials. This also raises the issue that the relative size of trials should in addition be considered in the context of the total number of patients with a given health condition. For COVID-19, at the time of this publication over 140,000,000 cases had been reported worldwide, but this number continues to rapidly change and there may be challenges for enrolment of COVID-19 patients specifically in cellular medicine trials.

### Overall COVID-19 results for cell therapy trials point to mostly low anticipated impact

For the combined dataset of both stem cell (including MSCs) and NK data from both databases, we found that of the 79 cell therapy trials for COVID-19, 67.1% were randomized, 25.3% were double blinded and 34.2% were placebo controlled. Only 18 of the 79 (22.8%) total trials satisfied the three categories of being randomized, double blinded and placebo controlled.

### Comparing trial design features when grouping by like phase

Since the trials in different groups of our database span a range of phases and phases impact the standards for design features, such as Phase I trials tending more broadly in the clinical trial arena to not include placebo controls, it was of interest to compare trials of the same phase. In order to compare like trials to like trials, we next analyzed the COVID- and non-COVID-related control groups when sorting by trials of the same phase.

Relatively few trials in either group were defined by the sponsors as ‘early Phase I’ or ‘Phase II/III’ (Table 2). Surprisingly, given the acute nature of the pandemic and the lack of existing, relevant preclinical data on cell therapies for COVID-19 since it is a new disease, only 14% of trials in the COVID-19-related group were Phase I, while 27.6% of trials in the control group were Phase I. In part this relatively low number of Phase I COVID-19 cellular medicine trials may be due to the literature on the general clinical safety of MSCs [15], allowing for regulators like the FDA to approve later phase trials as starting points. A third of COVID-19 trial listings were Phase I/II and another third were Phase II trials, while non-COVID-related trials had 26.7 and 19% listed as Phase I/II and Phase II trial status, respectively.

**Table 2.** Trial group phases

Trial type	Trials analyzed	Early Phase I	Phase I	Phase I/II	Phase II	Phase II/III	Phase III	Phase not specified
COVID-related trials	55	3 (5.5%)	8 (14.6%)	19 (34.6%)	19 (34.6%)	2 (3.6%)	1 (1.8%)	3 (5.6%)
Non-COVID control group (mean of three sets)	38.7	1.33 (3.4%)	10.7 (27.7%)	10.3 (26.6%)	7.33 (18.9%)	0.67 (1.7%)	3.7 (9.6%)	4.7 (12.1%)

Overall, non-COVID-19-related trials tended to be of an earlier phase, potentially contributing to their lower percentages having randomization, double blinding and use of placebo controls. However, non-COVID-19 trials did have more Phase III trials relatively speaking, but the actual mean number of such trials was a relatively low proportion (9.5%) of the control groups overall (Table 2).

When comparing Phase I COVID-19 trials to Phase I control non-COVID-19 trials, the trends we observed more broadly when analyzing the use of randomization, double blinding and placebo controls, held true (Table 3). The Phase I COVID-19 cellular medicine trials tended to employ these design features substantially more often than those unrelated to COVID-19. We also found this pattern to be the case when comparing the COVID-19- and non-COVID-19-related groups of only Phase II trials.

**Table 3.** Trial characteristics by phase

Trial phase	Trial type	n of trials	Randomized	Double blinded	Placebo controlled	All three
Phase I trials	COVID related	8	2 (25%)	2 (25%)	2 (25%)	2 (25%)
	Non-COVID related mean of three sets	10.67	3 (28.1%)	1.33 (12.5%)	0 (0%)	0 (0%)
Phase I/II trials	COVID related	19	13 (68.4%)	6 (31.6%)	9 (47.4%)	5 (26.3%)
	Non-COVID related mean of three sets	10.33	7.67 (74.2%)	2.33 (22.6%)	6 (58.1%)	2.33 (22.6%)
Phase II trials	COVID related	19	16 (84.2%)	9 (47.4%)	12 (63.2%)	9 (47.4%)
	Non-COVID related mean of three sets	7.33	4.33 (59.1%)	3 (40.9%)	2.33 (31.8%)	2.33 (31.8%)

In Phase I/II trials, the findings were somewhat distinct, however. The COVID-19 and non-COVID-19 trials were relatively more similar, with non-COVID-19 trials having modestly more use of randomization and placebo controls, but a little less of double blinding or of employment of all three design features (Table 3).

Because it was possible that MSC-based, non-COVID-related trial listings, in general and for arthritis, specifically include a large number of relatively low-quality listings, perhaps in some cases from for-profit stem cell clinics, reducing the apparent overall anticipated rigor of the control groups, we collected the names of all the sponsors in each group in our study. We then examined the sponsors’ names for their potential activities as for-profit stem cell clinics. In both the COVID-19 and arthritis groups, only four out of the 55 trials (~7.3%) in each case were sponsored by well-known stem cell clinics. In the no-disease specified, non-COVID control cohort only 2.4% of trials had sponsors that were clearly related to stem cell clinics. Thus, the presence of stem cell clinic sponsors in control groups does not explain the predicted somewhat higher impact of the cellular medicine COVID-19 group of trials.

### Geographic distribution of COVID-19 cellular medicine trials

**Figure 1.**
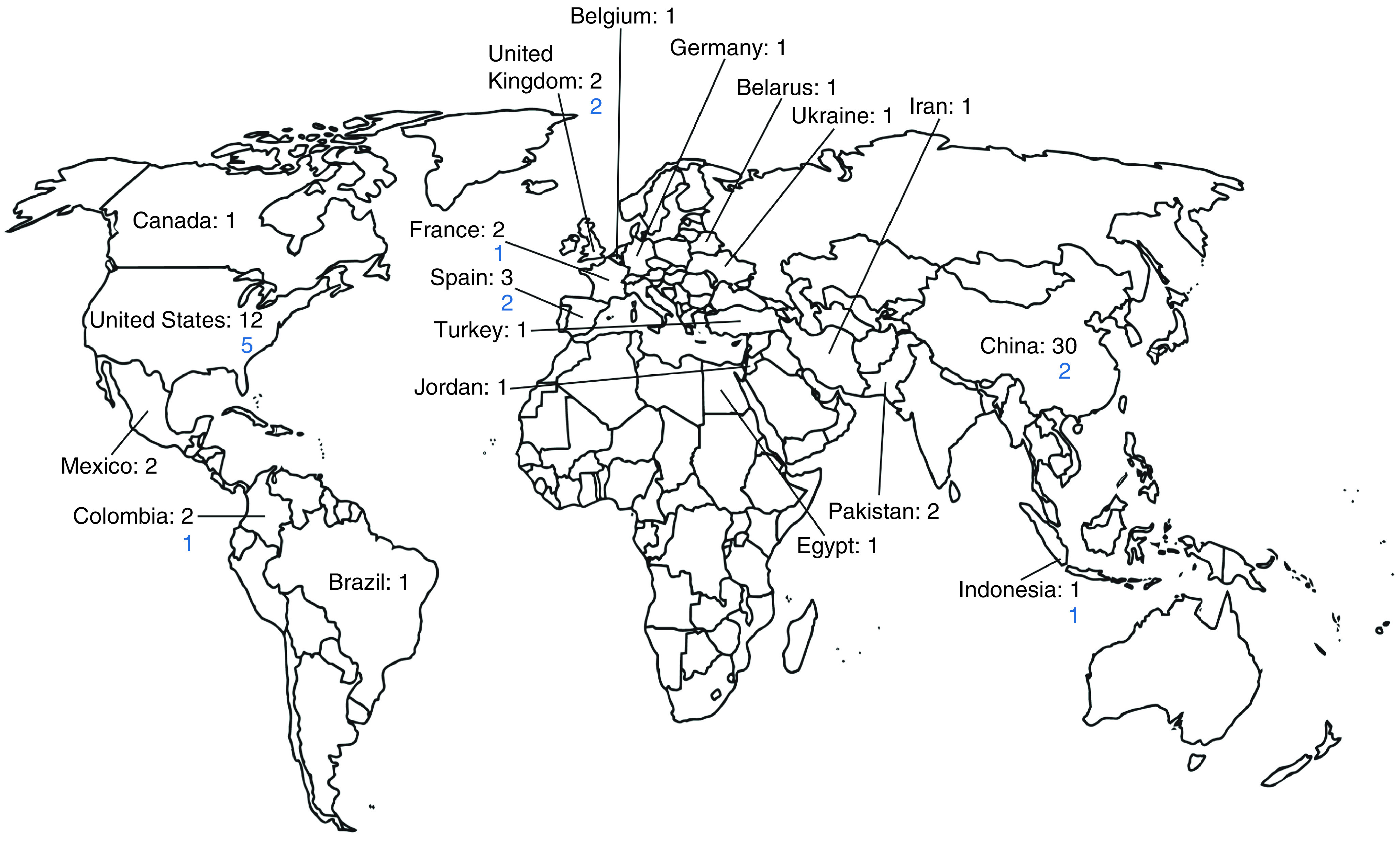
Map of geographic distribution of COVID-19 cellular therapy trials. The top number is the total number of trials in each country and the number underneath reflects the anticipated most impactful trials in that country.

Of the two registries, ClinicalTrials.gov had a more diverse geographic distribution compared with Chictr.org, which only had relevant clinical trials based in China. The majority of trial listings from ClinicalTrials.gov for both MSCs and NK cells were located in the USA and China. The geographic distribution of all cell therapy trials that we identified is shown in Figure 1 with that total data listed first, while the numbers of the anticipated most impactful trials (randomized, at least double blinded and placebo controlled) are indicated below. While we found that 18 trials were within the most impactful category, four of these had no location specified. China had the most COVID-19 cellular medicine trials in total with 30, but only two were within the most impactful category. The USA had 12 total trials, five of which were in the most impactful category. A diverse array of countries had at least one trial in our database.

## Discussion

Potential cellular therapy approaches to COVID-19 have generated major attention across the globe including from high-profile political figures, but we found the actual clinical trial landscape for cell therapies for COVID-19 mostly consisted of relatively small trials lacking the design features needed to conclusively determine safety and especially efficacy profiles. Media attention given to press releases, case reports and small (generally uncontrolled) trials may have given the wrong impression that this area is already established to be highly promising. Several problems could result from this disconnect including unproductive use of limited research resources due to duplicated research efforts. There may also be a reduction of patient populations available to participate in other potentially more promising types of COVID-19 trials. Overexuberance may lead to patients opting to take risks on unpromising cellular therapies for COVID-19 in the direct-to-consumer clinic market space or via self-experimentation, which also has been a concern in the ‘do-it-yourself’ COVID-19 vaccine area [16]. It is crucial for the cellular medicine and stem cell research fields to do everything possible to avoid even the appearance of weak science or pseudoscience, both related to COVID-19 and more generally, which is a real risk [17].

Unexpectedly, our data suggest that the pandemic does not appear to have led to less robust studies on cell therapies for COVID-19 as compared with our controls of cell therapy trials focused on arthritis or those on no one particular disease. Still, overall less than a quarter of COVID-19 cellular medicine trials were randomized, placebo-controlled and double-blinded studies, including less than a third of trials in the USA. This profile combined with the relatively small size of these clinical studies suggests to us that the data produced overall may be less instructive than one might have predicted given the 79 trials in this area that we identified overall at the time of our study.

There are some potential limitations to our study. As with any study based on registry data, sponsors in some cases may have listed inaccurate information or failed to list any information regarding key elements of their studies such as being randomized, double blinded and placebo controlled. We also found that a few trials contained seemingly contradictory information on blinding and masking. In the rare cases where individual trial listings mentioned having both single and double masking/blinding, we scored those trials as double blinded. We also only analyzed the intended trial designs listed in databases. Thus, this does not measure the actual ultimate success of trials, such as achieving complete enrolment of the desired number of patients and meeting goals in conducting trials as intended in other respects such as randomization, blinding and controlling.

In addition, there are challenges for interpretation of the characteristics of the COVID-19 trials versus our two control groups. It was possible, for instance, that MSC-based, non-COVID-related trial listings, in general and for arthritis, specifically included a large number of relatively low-quality listings, perhaps in many cases from for-profit, unproven stem cell clinics, reducing the apparent overall anticipated impact of the control groups. Unfortunately, ClinicalTrials.gov does not currently vet trial listing submissions for being legitimate trials and some stem cell clinic-sponsored trials may not be readily apparent as such. Even so, when we analyzed the names of all the sponsors in each group in our study, relatively few trials had known stem cell clinics as sponsors and the differences in the numbers of stem cell clinic sponsors did not appear to explain differences in anticipated impact of our different groups of trials.

Our findings also point to relatively low anticipated impact of cellular medicine trials more generally irrespective of COVID-19. One possibility is that as a relatively new field, cellular medicine still has a very large number of early phase, sometimes exploratory type trials that tend to be smaller and have fewer rigor-associated design features. Such trials are often more focused on safety than efficacy. As mentioned earlier, the ranks of cellular trials in registries overall may also include nontraditional studies by for-profit clinics that have less rigorous designs.

Notably, there were relatively few Phase I COVID-19 cellular therapy trials, which could be a result of the pressures from the pandemic to quickly try to look for efficacy and the extensive previous literature on MSC (the predominant product used) safety. However, it should be noted that there are little-to-no data on the safety of MSCs or other cells for COVID-19 specifically. In terms of trial phases, the trends we observed generally of COVID-19 trials having more rigor-associated design features held true when comparing trials just of the same phases (e.g., Phase I COVID-19 to Phase I non-COVID-19). While traditionally one would not necessarily expect a Phase I or even some Phase II trials to meet all of our criteria, some sponsors and other figures have publicly raised expectations that the interventions being tested will be effective treatments or cures of COVID-19.

In terms of trying to identify database-specific features of the COVID-19 trials, especially relevant to comparisons of the ClinicalTrials.gov and Chinese database trial profiles, the latter presented some challenges in terms of what search terms could be used productively. For example, searching for the single phrase ‘stem cells’ on the Chinese registry yielded no results, so a combined search of ‘stem’ and ‘cells’ as separate terms had to be conducted. While this presents potential difficulties for comparison of the results from the two databases, the COVID-19 studies identified on the Chinese registry consistently had much lower anticipated impact, which we believe is a *bona fide* difference in a general sense. However, this point is less definite given the distinct search terms used.

Our study highlights challenges related to trying to predict in advance the impact or rigor of relatively newly listed clinical trials. While we mainly used randomization, double blinding and employment of placebo controls as criteria related to predicted future rigor, we realize that these factors vary substantially based on trial phase. We also collected data on the size of trials, which is another potentially predictive impact-related factor, but did not find clear differences in trial sizes between the COVID-19 and the control non-COVID-19, no-specified disease group. Notably trials in the arthritis group were much larger, potentially reflecting the much longer period of time that arthritis cell therapy trials have been ongoing in general. Beyond randomization, double blinding, employment of placebo controls and the size of trials, we did not identify other strong candidate predictors of anticipated future impact of new or ongoing clinical trials where no data have been published in papers or on ClinicalTrials.gov or other registries.

Our study also points to challenges and possible lessons for regulators and researchers in the cellular therapy field more generally in terms of trying to predict impact or rigor of clinical trials. One advantage that regulators have in this regard is access to unpublished and otherwise confidential data. For instance, in the USA investigational new drug (IND) application-related data is generally confidential, but the FDA uses that data to evaluate trials for potential IND clearance. Presumably one element of this IND evaluation process is trying to predict the strength of trials and the preclinical data are crucial in that kind of process. While we did not have the benefit of access to such data, we believe our criteria were the best possible tools available to us even if there are limitations to such indicators as we discussed earlier. Our study and data suggest that other researchers seeking to evaluate specific groups of relatively new cellular therapy clinic trials may face challenges in trying to predict the impact. Even analysis of relatively more advanced clinical trials that have generated data may be hampered by the lack of deposition of such data on registries. The selection of control groups for studies of different groups of trials should be carefully considered and the potential presence of stem cell clinic listings on clinical trial registries should be examined.

We also found substantial diversity in the actual natures of the interventional offerings so that potential variable should also be considered by researchers. While we validated each study as correctly falling into the category of ‘cellular therapy’, some experimental products were actual cells, while others were derivatives of cells such as exosomes. Furthermore, the actual cells being used are quite diverse in some cases, even just for MSCs. Sources of these cells for the studies we identified included umbilical cord itself, cord blood, marrow, adipose tissue and dental pulp. MSCs from such diverse sources are likely to have some important, distinct properties. Both allogeneic and autologous preparations are also being studied. This kind of cellular product heterogeneity is also present in our control groups. Once trials produce actual data, the heterogeneity will also present difficulties for sponsors in comparing results between different trials. Even when many trials use the same broad terms like ‘MSC’ to describe their experimental therapies, researchers should keep in mind the possibility of wide heterogeneity in such trials.

The question arises as to whether it may be acceptable or even appropriate during a pandemic for some clinical trials to not necessarily meet the criteria that we employed. While it is unclear if there is a consensus within a new clinical area as with COVID-19 what trial designs in general would be appropriate and again the specific phase of trials will impact their use of certain trial design features, we favor an adherence to as highly rigorous standards as possible even during the pandemic, especially for later phase trials. While we did not find evidence that ‘COVID-19 exceptionalism’ [18] has lowered cell therapy trial standards at least as compared with other cell therapy trials, it will be difficult for most current trials in this area to generate conclusive results that are instructive for decision making in clinical care.

Initial published results in the area of cell therapies for COVID-19 are very limited. Dozens of articles on cellular therapies for COVID-19 continue to be published, but they consist mostly of review or perspectives pieces and case reports. A randomized, double-blinded, placebo-controlled study reporting indications of potential efficacy of umbilical cord cells for COVID-19 has generated attention recently [3], but it has some important limitations including small size (24 participants in total), which produced difficultly in balancing baseline characteristics of the control and interventional groups. Another more recently published umbilical cord MSC clinical trial for COVID-19 using a larger group of participants did not produce clear signals on safety and efficacy [19]. Overall, additional larger and generally more rigorously designed trials will be needed to conclusively determine the potential for efficacy and safety of cellular therapies for COVID-19 moving forward.

Looking ahead, analysis of the actual data that are collected from the existing pool of clinical trials documented in our study in coming years will be important. While such efforts may be further complicated by other factors such as the heterogeneity of COVID-19 patient populations and widespread concurrent treatment with other medicines such as steroids that are thought to work through very similar proposed immune-related mechanisms as the predominant cell therapy type being tested, MSCs [20], the data garnered are likely to provide unique and valuable information to the cellular therapy field and regulators.

## Conclusion

Overall, based on the late summer 2020 data, we predict relatively small collective clinical impact from the 79 cell therapy trials for COVID-19 we identified based on their design features. Perhaps as some existing trials advance to later phase clinical studies more high-level evidence will be generated. We hope that the few, relatively robustly designed cellular medicine trials for COVID-19 that we identified may produce conclusive, positive results in a time frame that is still relevant to the ongoing pandemic. More broadly, our study suggests that the cellular medicine field may benefit from considering developing phase-specific standards for trials to have the highest impact possible given the context of each trial.

**Summary points**

- The pandemic has sparked interest in trying to use cellular therapies such as stem cells to treat COVID-19.
- Prior to the pandemic relatively few clinical trials had produced data addressing the safety or potential efficacy of cellular therapies to treat diseases relevant to COVID-19 or other viral infections.
- Despite the small amount of existing relevant data, 79 new trials of various cellular therapies for COVID-19 were identified in ClinicalTrials.gov and the Chinese trial registry.
- The interventions being tested were diverse, but most often involved mesenchymal stem/stromal cells.
- Surprisingly, most of the new COVID trials were later than Phase I.
- The cellular therapy trials targeting COVID-19 were generally small (~40–50 participants) and relatively few met the three criteria used to assess anticipated potential clinical impact: randomization, at least double blinding and use of placebo control.
- Notably, even fewer cellular therapies trials in two control groups unrelated to COVID-19 met these criteria. Thus, the urgency of the pandemic may not have lowered standards for COVID-19 cellular therapy trials.
- Because more generally the particular phases of different clinic trials tend to have distinct levels of randomization, blinding and use of placebo control (e.g., Phase I trials employ these less often), we also analyzed control and COVID-19 groups of clinical trials by comparing subgroups of each by like phases, finding the same general trends as when we compared the groups unstratified by phase.
- Overall, this study predicts relatively little production of high-level data overall from COVID-19 cellular therapy trials, while also highlighting design challenges in cellular therapies more generally.
